# Design and characterization of a 3D‐printed axon‐mimetic phantom for diffusion MRI

**DOI:** 10.1002/mrm.28886

**Published:** 2021-06-30

**Authors:** Farah N. Mushtaha, Tristan K. Kuehn, Omar El‐Deeb, Seyed A. Rohani, Luke W. Helpard, John Moore, Hanif Ladak, Amanda Moehring, Corey A. Baron, Ali R. Khan

**Affiliations:** ^1^ Centre for Functional and Metabolic Mapping Robarts Research Institute Western University London Canada; ^2^ School of Biomedical Engineering Western University London Canada; ^3^ Department of Biology Western University London Canada; ^4^ Imaging Research Laboratories Robarts Research Institute Western University London Canada; ^5^ Department of Medical Biophysics Schulich School of Medicine and Dentistry Western University London Canada; ^6^ Department of Electrical and Computer Engineering Western University London Canada; ^7^ The Brain and Mind Institute Western University London Canada

**Keywords:** diffusion MRI, modeling and analysis, validation

## Abstract

**Purpose:**

To introduce and characterize inexpensive and easily produced 3D‐printed axon‐mimetic diffusion MRI phantoms in terms of pore geometry and diffusion kurtosis imaging metrics.

**Methods:**

Phantoms were 3D‐printed with a composite printing material that, after the dissolution of the polyvinyl alcohol, exhibits microscopic fibrous pores. Confocal microscopy and synchrotron phase‐contrast micro‐CT imaging were performed to visualize and assess the pore sizes. Diffusion MRI scans of four identical phantoms and phantoms with varying print parameters in water were performed at 9.4 T. Diffusion kurtosis imaging was fit to both data sets and used to assess the reproducibility between phantoms and effects of print parameters on diffusion kurtosis imaging metrics. Identical scans were performed 25 and 76 days later, to test their stability.

**Results:**

Segmentation of pores in three microscopy images yielded a mean, median, and SD of equivalent pore diameters of 7.57 μm, 3.51 μm, and 12.13 μm, respectively. Phantoms had T_1_/T_2_ = 2 seconds/180 ms, and those with identical parameters showed a low coefficient of variation (~10%) in mean diffusivity (1.38 × 10^−3^ mm^2^/s) and kurtosis (0.52) metrics and radial diffusivity (1.01 × 10^−3^ mm^2^/s) and kurtosis (1.13) metrics.

Printing temperature and speed had a small effect on diffusion kurtosis imaging metrics (< 16%), whereas infill density had a larger and more variable effect (> 16%). The stability analysis showed small changes over 2.5 months (< 7%).

**Conclusion:**

Three‐dimension‐printed axon‐mimetic phantoms can mimic the fibrous structure of axon bundles on a microscopic scale, serving as complex, anisotropic diffusion MRI phantoms.

## 1 INTRODUCTION

Diffusion MRI (dMRI) produces images by sensitizing the MRI signal to the random motion of water molecules on a micrometer scale. Models and representations of dMRI have been developed to describe the dMRI signal and produce metrics that are related to the microstructure and connectivity of the brain. To be clinically useful, the accuracy and reliability of the dMRI‐based model and representation parameters should be validated, but validation is difficult because there is typically no in vivo ground truth for comparison.

A number of techniques have been used to produce dMRI ground truths,^1^, ^2^ including numerical phantoms that simulate scan data,^3^, ^4^ histology of brain samples scanned ex vivo^5^, ^6^ or in vivo before extraction,^7^, ^8^, ^9^ and physical phantoms with separately characterized microstructure.^10^, ^11^ Numerical phantoms can use analytic models of diffusion in substrates composed of well‐defined compartments,^12^ or Monte Carlo simulations of diffusion using arbitrarily complex and realistic mesh‐based substrates.^13^, ^14^, ^15^ Numerical phantoms allow precise experimental control and increasingly realistic substrates, but realistic Monte Carlo simulations demand significant computational resources, which limits the possible volume of simulated substrates. Analyzing histological sections of previously scanned brain tissue^16^ provides data directly from a real brain, but histology is time‐consuming and costly, covers a limited region of interest, and is difficult to register to a dMRI scan, especially when the scan was performed in vivo. Microstructural changes due to fixation and histological preparation limit the correspondence between these studies and in vivo imaging. Physical phantoms are artificial objects designed to mimic the diffusion characteristics of the brain, ideally producing dMRI scan data similar to that seen from real brains. Physical phantoms occupy a middle ground between the two ends of a spectrum defined by numerical and ex vivo studies: They produce real scan data with well‐known microstructural ground truth, but are not as customizable as numerical phantoms or as true to the structure of the brain as ex vivo samples.

Several types of physical phantoms have been previously proposed. Glass capillaries^17^ provide reliably anisotropic diffusion in a pattern that is straightforward to characterize, but cannot mimic some complex geometric fiber configurations that are observed in the brain. Plain (solid/nonhollow) fibers consisting of a variety of materials are available on the market^18^ with axon‐scale diameters to mimic hindered diffusion between axons,^19^, ^20^, ^21^ but are difficult to arrange with the geometric complexity of some brain regions. Extruded or electrospun hollow fibers^22^ mimic axonal diffusion patterns well, but require specialized equipment like high‐voltage power supplies^23^ or melt‐spinning extruders^24^ to produce. As such, the development of dMRI phantoms involves trade‐offs between the cost of materials and equipment, ease of production, the ability to achieve accurate brain‐mimetic microstructure, and geometric complexity. Existing procedures to produce a physical phantom with biologically plausible microstructure and geometric complexity require specialized equipment and/or are time‐consuming, so an alternative that is both adequately brain‐mimetic and easy to produce is needed.

**FIGURE 1:**
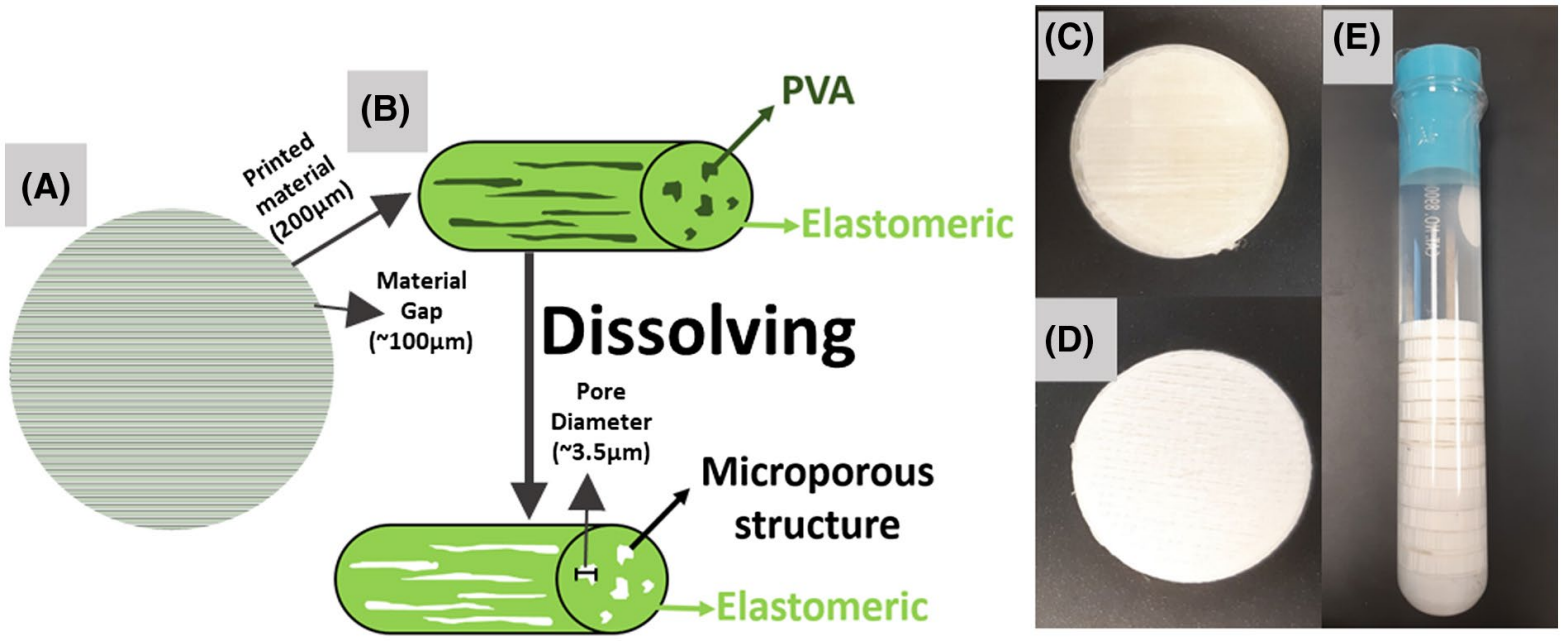
Illustration of 3D‐printed axon mimetic (3AM) phantom microstructure and photos of printed phantoms. The cylinders in (B) represent a single line of printed material showing the printed material and the gaps in between the lines. The disks in (C)‐(E) consist of hundreds of individual printed lines (as illustrated in [A]) that are printed along the horizontal direction in (A,C,D) and perpendicular to the long axis of the test tube in (E). A, The 3AM phantom printing schematic, in which the material is printed along parallel lines. B, In each printed line, polyvinyl alcohol (PVA) dissolves away when placed in water, leaving the microporous structure. C, The 3AM phantom before dissolving. D, The 3AM phantom after dissolving. E, Dissolved 3AM phantoms stacked in a test tube with water that are ready for imaging

We propose the use of fused deposition modeling (FDM) 3D printing^25^ as an inexpensive and flexible means of producing dMRI phantoms. To use FDM to produce 3D‐printed axon mimetic (3AM) phantoms, we propose the use of a dual‐component “porous filament” material.^26^ In this work, we used GEL‐LAY (LAY Filaments, Cologne, Germany), which consists of a hydrophobic elastomeric matrix infused with pockets of polyvinyl alcohol (PVA). When 3D‐printed, the PVA forms long fibers within each line of printed composite material. The PVA is water‐soluble, so when this 3D‐printed material is immersed in water, the PVA fibers dissolve, leaving behind microscopic fibrous pores, as illustrated in Figure 1B. The anisotropic structure of these fibrous pores are similar to the anisotropic shape of axonal fibers and restrict diffusion in a similar way, potentially allowing 3D printed porous filaments to form the basis of a phantom that, due to the freedom to print along arbitrary directions, can potentially characterize the response of dMRI representations^27^ and models^28^ relative to fiber bending and crossing.

In this study, we use both fluorescent microscopy and synchrotron phase‐contrast micro‐CT, a novel technique that allows exceptionally fine‐resolution 3D visualization of soft structures, to characterize and visualize the microstructure of 3AM phantoms. We examine the effects of FDM printing parameters on dMRI acquired in the phantoms, and assess the reproducibility and stability over time of 3AM phantoms’ dMRI characteristics. We also construct an analytic model of diffusion in 3AM phantoms based on the microscopy and micro‐CT imaging and compare simulated diffusion signal from the analytic model to measured diffusion signal, assessing the accuracy of our characterization of 3AM phantoms’ microstructure.

## 2 METHODS

### 2.1 Phantom preparation

An open protocol for producing 3AM phantoms has been developed and released as part of an Open Science Framework project (available in the Supporting Information and hosted at osf.io/zrsp6). Required materials include an FDM 3D printer, a dual‐component “porous filament” material, a vacuum chamber, and a set of watertight containers. The protocol was used to produce all phantoms for this study.

All phantoms were designed using the open‐source *Ultimaker C*ura software and were printed with an Ultimaker 3 Extended 3D printer (Ultimaker, Geldermalsen, the Netherlands) loaded with GEL‐LAY filament. Unless otherwise noted, phantoms were printed with printing parameters recommended by the material vendor (Table 1), and the same pattern of parallel lines of material in each layer.

**TABLE 1:** Parameters used to print the phantoms

Printing parameter	Low setting	Nominal setting	High setting
Printing temperature (°C)	215	225	235
Printing speed (mm/s)	20	30	40
Layer thickness (mm)	0.06	0.1	0.14
Infill density (%)	75	100	—

After printing, phantoms to undergo dMRI scanning were immersed in 1 L of room‐temperature tap water (~23°C) for 168 hours, then 20 mL of surfactant was added to decrease surface tension and allow the water to more easily enter the pores. The container was placed in a vacuum chamber at 1 bar for 48 hours to remove air bubbles. Finally, the phantoms were stacked in a test tube with distilled water for imaging (Figure 1C,D). Phantoms that undergo dMRI are kept in water at all times after preparation.

### 2.2 Microscopy

Three block‐shaped 3AM phantoms with dimensions 25 × 15 × 35 mm were printed with vendor‐recommended printing parameters. The blocks were immersed in water for 18 hours to soften by allowing a small portion of the PVA to dissolve, allowing them to reach the appropriate hardness level for slicing. Afterward, the blocks were sliced into 50‐μm slices in the plane transverse to the long axis of the pores using a Shandon Finesse ME+ microtome (Thermo Fisher Scientific, Waltham, MA). The slices were immersed in deionized water for 3 hours to allow the remaining PVA to fully dissolve. They were then immersed in diluted rhodamine beta (Rhodamine B) and placed in a vacuum chamber at 1 bar for 20 minutes to infuse the dye into the slices and eliminate air bubbles. The slices were then mounted on positive microscope slides.

Confocal microscopy was performed to obtain high‐resolution images using a Leica SP5 laser system microscope with a 40× oil‐immersion objective lens. Z‐stacks of phantom slices were acquired using 1.0‐μm step size for axial ranges between 5 and 17 μm. Using the *MATLAB* image processing toolbox (Natick MA), three averaged z‐stack confocal microscopy images were converted to grayscale (one from each block), then an adaptive thresholding technique^29^ was used to compute segmentation of each image. Finally, assuming each region identified as a pore was approximately circular, the equivalent radius of each region identified as a pore was calculated from its area A according to the formula $r_{\mathrm{Eq}}=\sqrt{A/\pi }.$

### 2.3 Synchrotron phase‐contrast micro‐CT

A cylindrical phantom with a diameter of 5 mm and height of 4.5 mm was 3D‐printed, immersed in water for 2 days, and allowed to dry to improve CT contrast between the pores and the lattice. A propagation‐based phase‐contrast micro‐CT scan was then performed in air at the Biomedical Imaging and Therapy beamline (05ID‐2) at the Canadian Light Source (Saskatoon, Canada) using an energy of 30 keV, 3000 projections over 180°, 150‐ms exposure time and 700‐ms time per projection, total scan time of 1 hour, FOV of 4096 × 4096 × 3456 μm^3^ (L × W × H), and an effective isotropic pixel size of 1.65 μm.

The micro‐CT volume was segmented in two steps. First, the volume was rotated to align the lines of material with the axes of the volume, cropped to remove the edges of the sample, and down‐sampled to one‐eighth of the initial resolution. The air‐filled regions between lines of material were then segmented in the preprocessed volume by thresholding the SD computed in local 3 × 3 × 3 windows. The resulting mask was then up‐sampled back to the source resolution.

To segment the pores in the micro‐CT volume, a selection of 17 x‐z slices 80 μm apart was chosen, covering a 1.28‐mm section of the middle of the phantom. The air‐filled region of each slice was masked out using the segmentation produced previously, and each resulting 2D image was segmented using an adaptive thresholding technique^29^ from the *MATLAB* image processing toolbox. Assuming each region identified as a pore was approximately circular, the equivalent radius of each region identified as a pore was then calculated from its area A according to the formula $r_{\mathrm{Eq}}=\sqrt{A/\pi }.$

### 2.4 Magnetic resonance imaging scanning

Phantoms to undergo dMRI scanning were all scanned with the same parameters. Diffusion MRI was implemented with a 9.4T Bruker small animal scanner using a pulsed gradient spin‐echo single‐shot EPI sequence with 120 and 60 directions at b = 2000 and 1000 s/mm^2^, respectively, 20 averages at b = 0 s/mm^2^, diffusion gradient lobe duration (δ) of 4.06 ms, spacing between gradient lobes (Δ) of 13.1 ms, gradient magnitudes calculated to achieve the intended b‐values, TE/TR = 37/2500 ms, FOV = 200 × 200 mm^2^, 0.7‐mm isotropic in‐plane resolution, and one 3‐mm axial slice per phantom (8.5‐minute scan time) for a total of four phantoms per scan. Two scans were required to acquire data for a total of eight phantoms for the study. The test tubes were repositioned between scans.

For every resulting diffusion‐weighted image, DiPy^30^ was used to compute diffusion kurtosis imaging (DKI) metrics^31^, ^32^ with a weighted ordinary least‐squares approach^33^ at each voxel in a region of interest manually drawn to avoid air bubbles in the phantoms.

A T_1_‐mapping rapid acquisition relaxation enhancement scan and a T_2_‐mapping multislice‐multi‐echo scan were performed on a set of eight phantoms with different 3D‐print parameters (section 2.6). Both scans were implemented with a 9.4T Bruker small animal scanner, FOV = 40 × 40 mm^2^, 0.3125‐mm isotropic in‐plane resolution, and one 3‐mm axial slice per phantom. Each slice was centered in a single phantom, mitigating the potential for partial volume effects between phantoms. The T_2_ mapping scan was performed with 16 echoes having TE ranging from 6.5 to 104 ms and TR = 2000 ms. The T_1_ mapping scan was performed with TE = 5.7 ms, TRs of 500, 1000, 1500, 2000, and 3000 ms, and echo train length = 2.

The T_2_ was fit by performing a simple linear regression of the signal in each voxel to the equation $\ln\left(S\right)=\ln\left(S_{0}\right)‐\left(TE/T2\right)$, where S is the measured signal; S_0_ is the theoretical signal at TE = 0 ms; and T_2_ is the material’s T_2_ at that voxel. The T_1_ was fit by performing a least‐squares fit of the signal in each voxel to the equation $S=C\left(1‐\exp\left(‐TR/T1\right)\right)$, where S is the measured signal; C is a constant that depends on the TE, T_2_^*^, and proton density; and T_1_ is the material’s T_1_ at that voxel. The least‐squares fit was performed with SciPy’s “curve_fit” method^34^ using the Trust Region Reflective method.^35^ To assess typical T_1_ and T_2_ values in 3AM phantoms, the mean and SD T_1_ and T_2_ across all pixels in the region of interest were calculated in the phantom produced with the nominal print parameters recommended by the manufacturer. The mean S_0_ from the T_2_ fit was also estimated across all voxels in the scan with an estimated T_2_ over 1 second as a proxy for the proton density of water.

### 2.5 Phantoms for assessing the reproducibility of dMRI metrics

Four identical cylindrical 3AM phantoms were prepared with nominal printing parameters following the procedure outlined in section 2.1. The phantoms were scanned with a total of four axial slices (one slice per phantom), and DKI was fit to the scan data, all according to the imaging protocol outlined in section 2.4. The coefficient of variation across the mean parameter values from the four phantoms was calculated for each DKI metric, by dividing the SD of the four values by the mean of the four values, to assess the consistency of each metric across phantoms produced under identical conditions.

### 2.6 Phantoms for assessing stability over time and the effect of print parameters on dMRI

Eight cylindrical 3AM phantoms were produced according to the 3AM phantom protocol outlined in section 2.1, each with a width of 22 mm and height of 4.4 mm, consisting of 44 layers of parallel lines.

Four FDM print parameters were altered across different phantoms to test their effects on the 3AM phantom’s microstructure. The four altered parameters include the temperature at which the phantoms are printed, the speed of printhead travel during the 3D print, the thickness of each layer printed in the phantom, and the infill density, which refers to the proportion of each layer taken up by the material, and is altered by changing the distance between adjacent lines of material within each layer. One phantom was printed with the nominal print parameters recommended by the substrate manufacturer, and for each parameter, one phantom was printed with that parameter lower than nominal, and one phantom with that parameter higher than nominal (where applicable), with all other parameters kept at their nominal value, as summarized in Table 1.

The eight phantoms were scanned with four axial slices and a scan time of 8.5 minutes per each of two dMRI scans with a total of eight axial slices to cover the test tube and DKI was fit to the data, all according to the protocol outlined in section 2.4. The variation in each DKI metric was then assessed across the phantoms with each difference in print parameter.

To investigate the phantoms’ stability over longer time periods, identical dMRI scans and model fitting procedures were performed 25 and 76 days later, and the variations in each DKI metric in the phantom produced with nominal parameters across time was assessed.

### 2.7 Simulation

Camino^12^, ^36^, ^37^ was used to construct an analytic diffusion model consisting of two compartments specified by Panagiotaki et al^12^: a free water (“Ball”) compartment and a compartment consisting of water within parallel cylinders with radii distributed according to a gamma distribution (“GDRCylinders”). The only parameter of the “ball” compartment is the water’s diffusivity, which we set at 2.1 × 10^−3^ mm^2^/s, the diffusivity of free water at room temperature. The “GDRCylinders” compartment has five parameters: The diffusivity was also set to 2.1 × 10^−3^ mm^2^/s; the shape (a) and scale (b) parameters of the gamma distribution were determined using a maximum‐likelihood fit of the equivalent pore diameters measured from the segmented microscopy images. The orientation parameters θ and ɸ, which define the relative orientation between compartments, were arbitrarily set to zero because the ball component has no directionality. The final parameter of the simulations is the proportion of water within the ball compartment, which we estimated from the volume fraction of space between lines of material in the micro‐CT volume. We simulated a dMRI scan of our analytic model using the same experimental scan parameters we used for the phantoms and fit DKI to the simulated signal using DiPy, as we did for the experimental signal.

## 3 RESULTS

### 3.1 Microscopy

**FIGURE 2:**
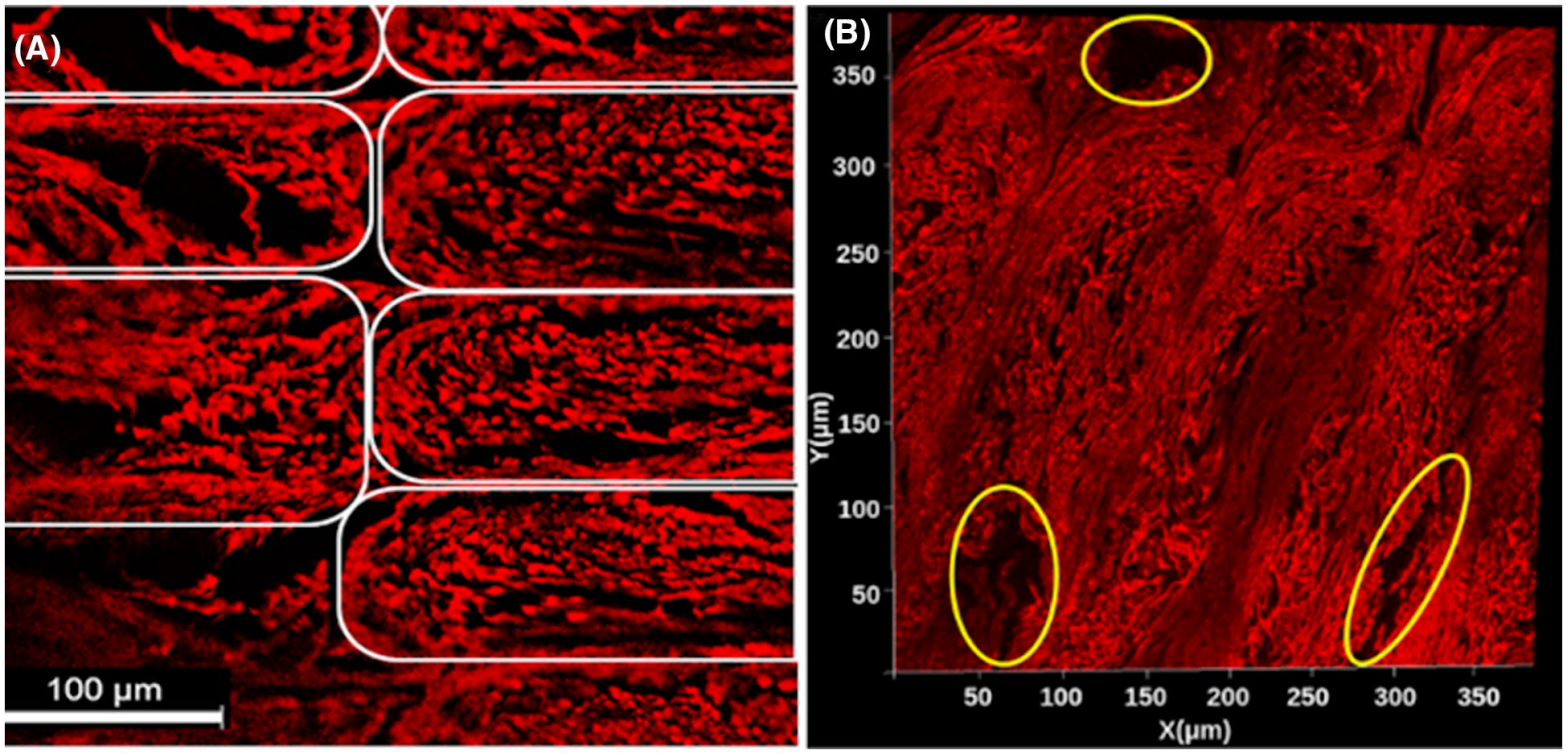
A, Confocal microscopy z‐stack image of a stained cross‐sectional phantom sample, averaged across slices. Elastomeric matrix (red) and pores (black) are visible. Each white outline indicates an individual line of material, as depicted in Figure 1B. B, The 2D projection of a 3D microscopy volume acquired with confocal microscopy. Regions shown in red are the matrix of the 3AM phantom that is composed of elastomer, while the black regions are pores. Outlined in yellow are larger pores caused by the printing pattern of the phantom. In both (A) and (B), the image plane is perpendicular to the long axis of the pores

**FIGURE 3:**
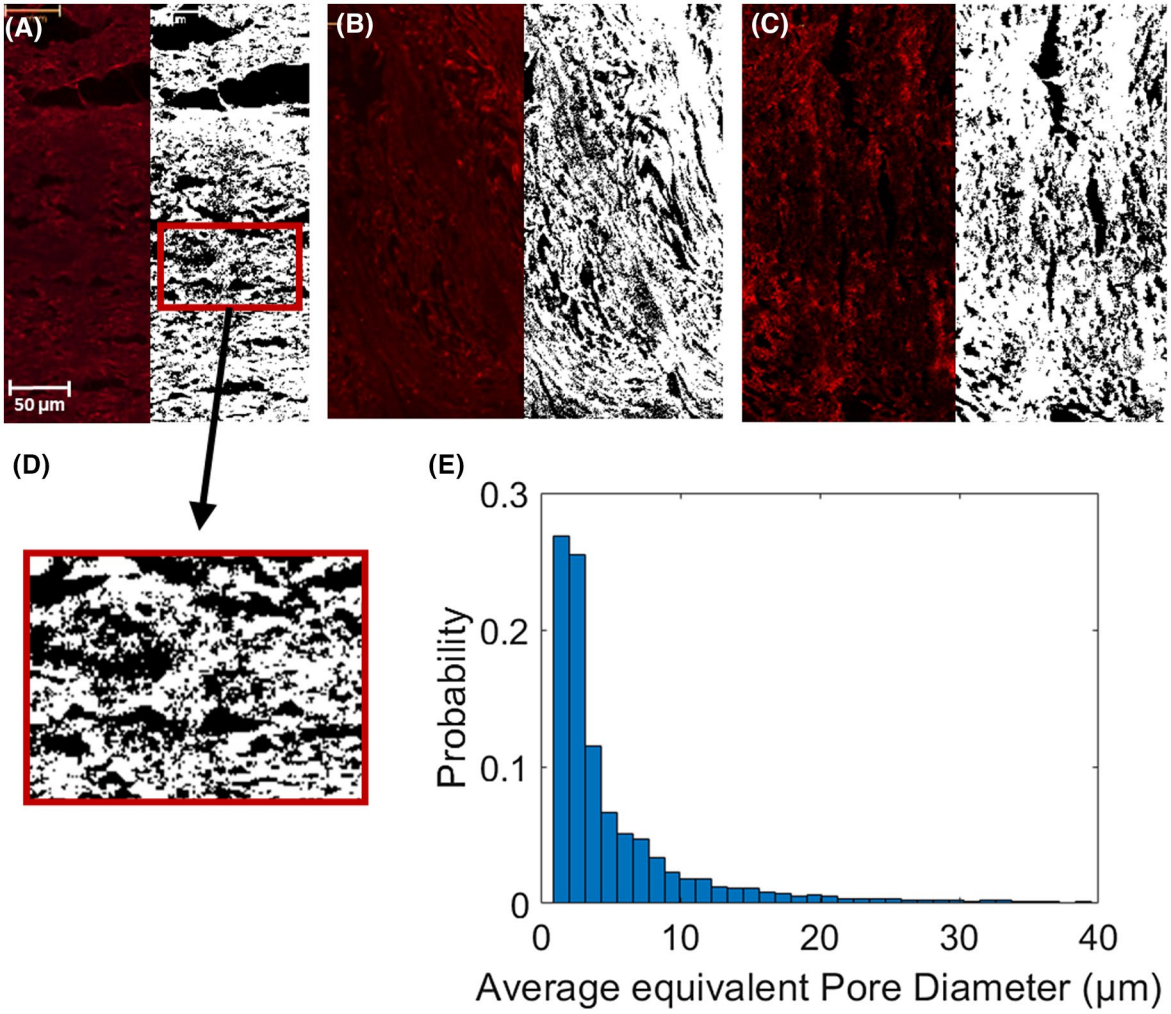
A‐C, Confocal microscopy image before (left) and after (right) performing pore segmentation. Segmented pores are shown in black. D, Zoomed‐in picture of segmented pores on (A). E, Normalized histogram of pore equivalent diameters from the three segmentations in (A)‐(C) (total *N* = 10 762)

Confocal microscopy images of slices in the plane transverse to the long axis of the pores revealed two types of pores present in the phantoms: larger pores (70‐150 μm in diameter) that are believed to have been created by material tearing during the microscopy preparation process and gaps being left between lines of material (Figure 2), and smaller pores (1‐30 μm in diameter) created by the dissolving of PVA fibers (Figure 3). The images also clearly showed the larger‐scale arrangement of each line of material deposited during the 3D‐printed process.

The segmented pores from three averaged z‐stack confocal microscopy images had a mean equivalent diameter of 7.57 μm, a median equivalent diameter of 3.51 μm, and a SD of 12.13 μm (Figure 3).

### 3.2 Micro‐CT

**FIGURE 4:**
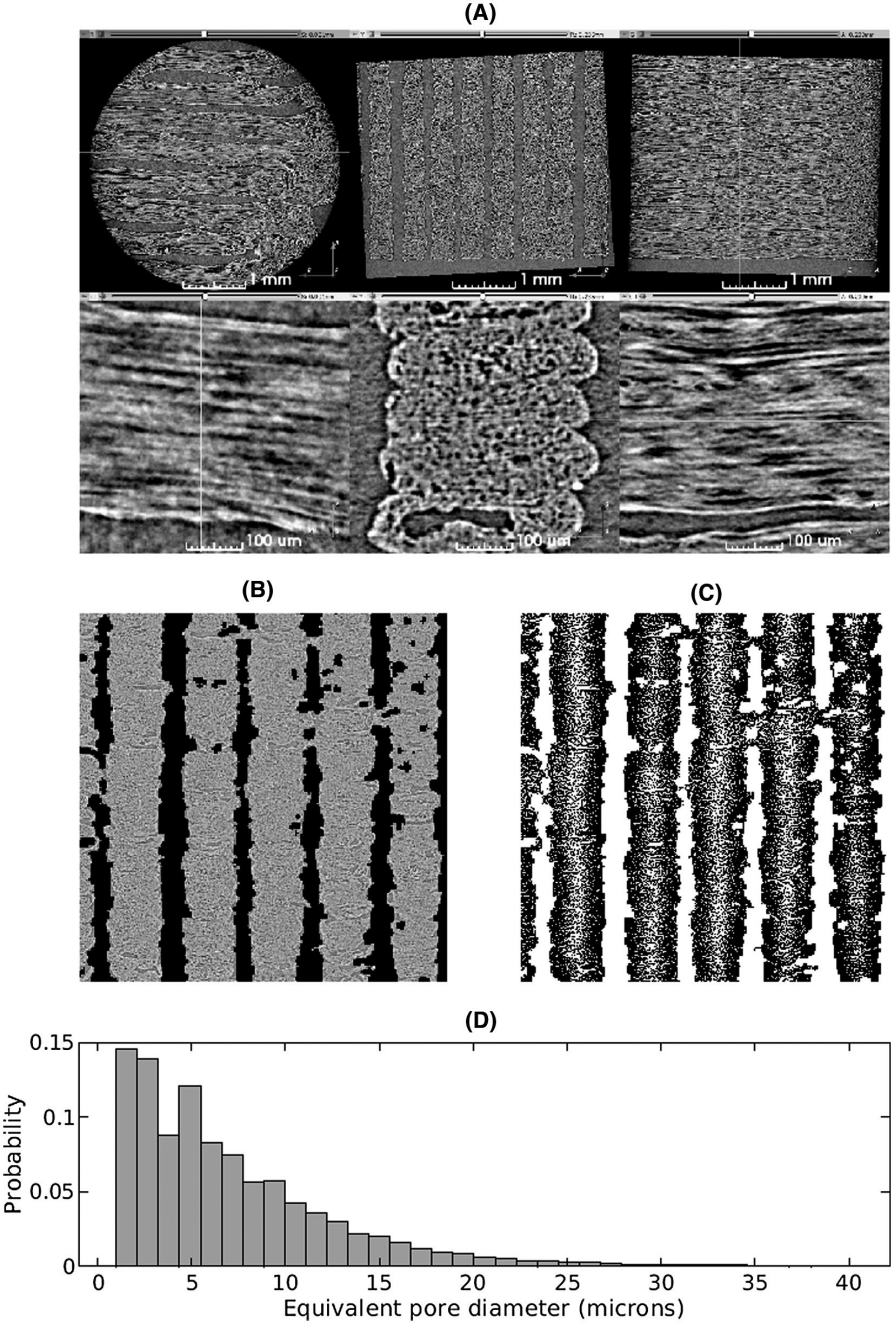
A, Synchrotron micro‐CT scan data at two zoom levels, transformed to align the lines of material with the viewing planes. Upper row: View of the entire‐scan region of interest. Lower row: Detailed view of a short length of five stacked lines of material. The direction of print‐head motion was left–right in the left‐most and right‐most columns, and perpendicular to the image in the center column. The remaining panels show a micro‐CT slice with air‐filled regions masked out (B), segmentation results with pores shown in white (C), and a histogram of pore equivalent diameters from 17 slices (D)

The propagation‐based phase‐contrast micro‐CT image showed anisotropic pores that run parallel to the primary travel direction of the 3D print head (Figure 4A), supporting the findings of the confocal microscopy. These pores had typical diameters on the order of 10 microns and typical lengths in the hundreds of microns.

The segmented pores from 17 slices of the micro‐CT volume had a mean equivalent diameter of 8.77 μm, a median equivalent diameter of 5.71 μm, and the SD of the sample of equivalent pore diameters was 21.42 μm (Figure 4B‐D).

### 3.3 Nominal phantom characteristics and reproducibility

**FIGURE 5:**
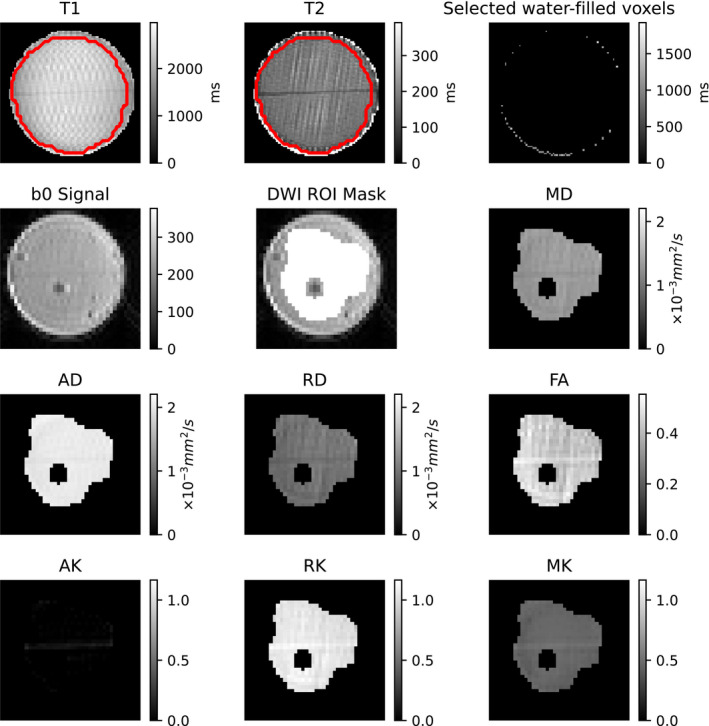
Examples of the produced maps in the nominal phantom. Top row: T_1_ and T_2_ maps with region‐of‐interest (ROI) mask outline superimposed (colormap limits drawn from within the ROI), and selected water‐filled voxels between the phantom and test tube. Second row: Example of non–diffusion‐weighted image (DWI), ROI mask superimposed on the non‐DWI, and mean diffusivity (MD). Third and fourth rows: Diffusion kurtosis imaging (DKI) diffusivity metrics. Abbreviations: AD, axial diffusivity axial diffusivity; AK, axial kurtosis; RD, radial diffusivity; RK, radial kurtosis

Examples of each image and metric map captured, with the region of interest used, are shown in Figure 5. Some material leaked as the 3D print head traveled to reset between each printed layer, leaving a line of fibrous pores oriented nearly perpendicular to the intended pores. This line of leaked material is particularly prominent on the T_2_ and axial kurtosis (AK) maps. This line only affected a small number of voxels relative to the size of the mask, and likely had only a small effect on net measured parameters.

The mean ± SD T_1_ and T_2_ measured in a phantom printed with nominal printing parameters were 2,309 ± 250.47 ms and 183 ± 28 ms, respectively. The mean S_0_ in the nominal phantom was 81.61, and the mean S_0_ in the other voxels with T_2_ > 1 second was 130.3, suggesting that the proton density of the phantom is about 63% of the proton density of water. Considering the approximate 100‐μm gaps between each 300 μm of porous material observed from micro‐CT, the proton density of the porous material is about 50% of pure water. The artifacts in the T_1_ map are likely due to vibrations of the small test tube. Although this causes artifacts for the multishot sequence used for the T_1_ map, the effect on the dMRI acquisition that used single‐shot EPI was likely negligible.

The mean value of each DKI metric was calculated in each of the four nominal phantoms, then the mean and SD for each DKI metric was calculated across those four values, as summarized in Table 2. The axial diffusivity (AD) is close to the diffusivity of pure water at room temperature (0.0022 mm^2^/s), and the radial diffusivity (RD) is about half the AD. The AK is close to zero, suggesting little diffusion restriction in the direction parallel to the pores. The highest coefficient of variation across the four nominal phantoms was 15.00% for AK, and the rest were lower than 8%.

**TABLE 2:** Mean diffusion MRI metrics from four nominal phantoms and their coefficient of variation

Metric	Mean value	SD	Coefficient of variation (%)
AD (×10^−3^ mm^2^/s)	2.13	0.05	2.41
RD (×10^−3^ mm^2^/s)	1.01	0.08	7.57
MD (×10^−3^ mm^2^/s)	1.38	0.07	4.88
FA	0.46	0.03	6.58
AK	0.07	0.01	15.00
RK	1.13	0.07	5.78
MK	0.52	0.02	3.62

### 3.4 Metric variation with print parameters

**FIGURE 6:**
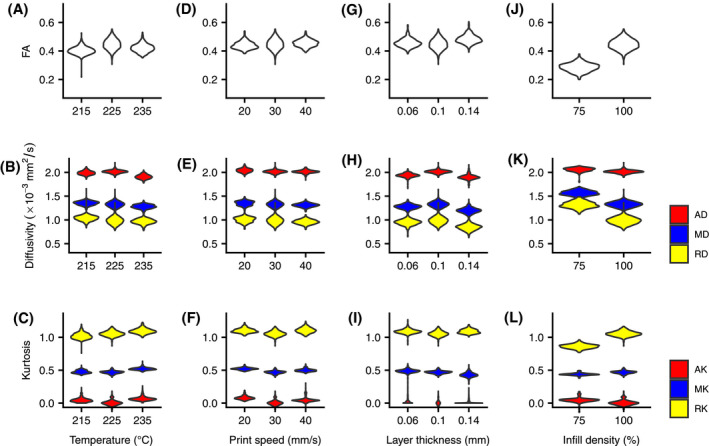
Mean fractional anisotropy (FA), diffusivities, and kurtosis with different phantom printing parameters. A‐C, Different metrics with different printing temperatures. D‐F, Different metrics with different printing speeds. G‐I, Different metrics with different layer thicknesses. J‐L, Different metrics with different infill densities. Violin plots correspond to the distribution of a metric over all the voxels in a phantom

Changes in the mean value of each metric with every print parameter were small for every print parameter except infill density (Figure 6). Excluding infill density, the difference from the nominal case was less than 16% for all metrics except AK, which had very high percent differences in some cases due to mean values close to zero. An increase in infill density of the phantom resulted in an increase in fractional anisotropy and a decrease in radial diffusivity.

### 3.5 Metric stability over time

**FIGURE 7:**
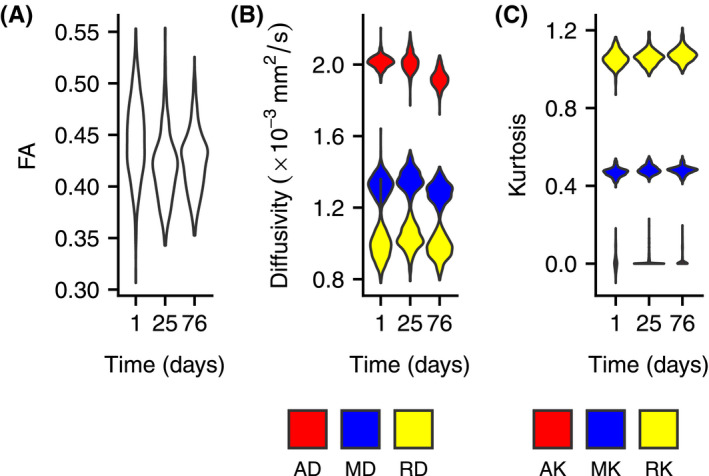
A, Mean FA of a nominal phantom over 76 days. B, The MD of a nominal phantom over 76 days. C, Mean kurtosis of a nominal phantom over 76 days. Violin plots correspond to the distribution of a metric over all the voxels in a phantom

Over the 76 days of study, only small differences in mean value were observed for all metrics (< 7%, except for AK due to some very small values of AK), as seen in Figure 7.

### 3.6 Simulation

We estimated that 34% of the micro‐CT volume consisted of open space between lines of material, so we set the proportion of water within the “ball” compartment of the analytic model to 0.34. The maximum likelihood fit of the gamma distribution to the array of equivalent pore diameters segmented from the microscopy images yielded the shape parameter a = 1.18 and the scale parameter b = 6.39 μm, which we used in the “GDRCylinders” compartment of the analytic model.

**FIGURE 8:**
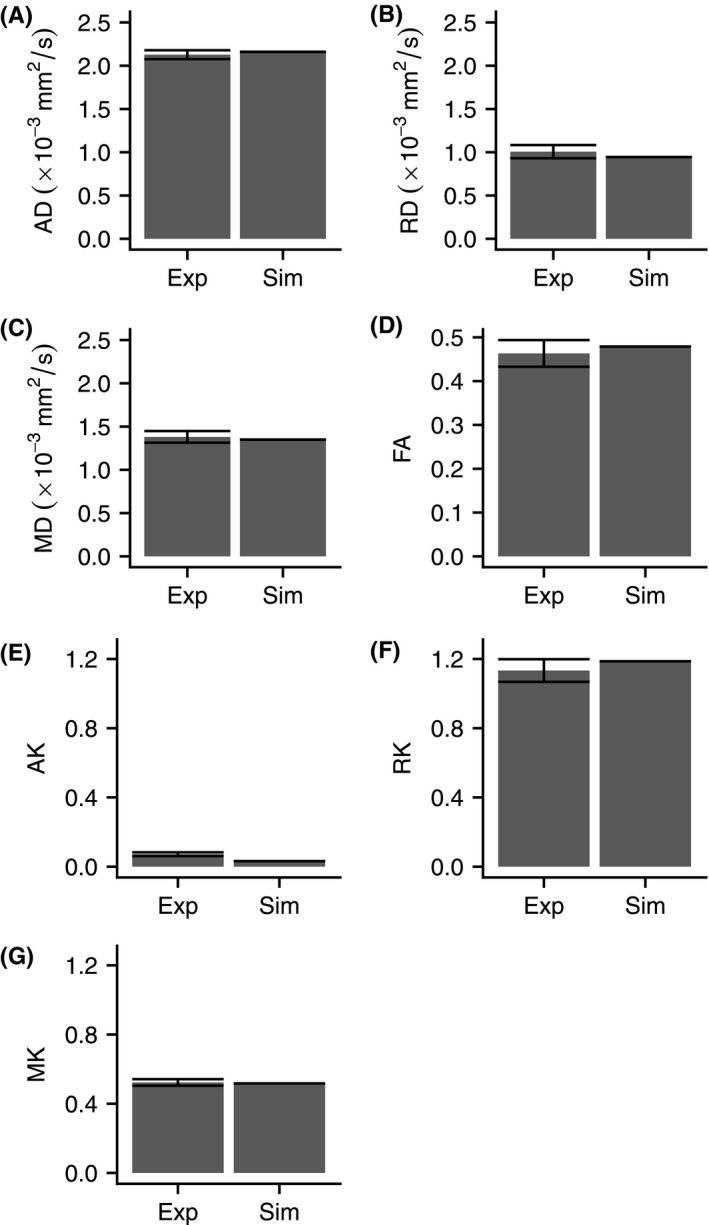
Mean experimentally observed (Exp) and simulated (Sim) DKI metrics. Error bars show one SD above and below the mean experimentally observed value. The experimentally observed values correspond to the values in Table 2, and the simulated metrics originate from fitting to noise‐free signals, so there is one value and no error bar associated with each metric: AD (A), RD (B), MD (C), FA (D), AK (E), RK (F), and mean kurtosis (MK) (G)

Fitting DKI to the simulated signal yielded a radial diffusivity of 0.944 × 10^−3^ mm^2^/s and radial kurtosis of 1.19. The simulated RD and RK have absolute differences of 0.066 × 10^−3^ mm^2^/s and 0.06 compared with the empirically observed mean RD and RK, respectively (Figure 8).

## 4 DISCUSSION

### 4.1 Phantom versus human microstructural fiber geometry

Microscopy images and synchrotron micro‐CT images both indicate the presence of densely packed fibrous pores within a 3D‐printed composite material containing PVA within an elastomeric matrix. It is difficult to characterize the microscopic properties of an entire phantom, and representative slices must be used; nevertheless, the close correspondence between simulated and measured diffusion behavior suggests that the microscopy images resolve most of the phantoms’ pores and that the pores are approximated well as straight cylinders.

A potential limitation of the pore‐size gamma distribution fitting is that primarily the tails of the histogram were used in the fitting. However, the smallest axons that were missed due to resolution limits only contribute to 0.11% of the total water volume, assuming the fitted gamma distribution parameters, and this error likely had a small effect on diffusion parameters estimated from simulation. Although the pores were generally not circular, the agreement of our simulations with the experimentally observed values suggests that using an effective diameter based on the cross‐sectional area may be an appropriate simplifying assumption. However, a notable limitation of these phantoms is that because they are composed of fibrous pores in a solid matrix, they have no analog to the extra‐axonal hindering of diffusion observed in the brain.

Another potential limitation of the phantoms is that the under‐extrusion of the porous material during the 3D‐printing process results in narrower lines of material than intended, leaving roughly 100‐μm‐wide gaps between lines of material in each layer (for 100% nominal infill density; gaps ~200 μm for 75% infill density). Once the phantoms were immersed in water, water filled these gaps, leading to about 34% of the phantom consisting of free water. This free water behaves analogously to a partial volume of CSF within the voxel. Adjusting additional 3D print parameters, for example, by using a wider nozzle, may reduce the under‐extrusion and eliminate the gaps. Alternatively, the under‐extrusion might be mitigated by using a nominal infill density greater than 100% to achieve an effective infill density of 100% in the presence of under‐extrusion.

With a mean equivalent diameter of 7.56 μm and median equivalent diameter of 3.51 μm (Figure 3), these fibrous pores are larger than typical axons.^38^, ^39^, ^40^ This could have a considerable impact on using these phantoms to validate models that are sensitive to pore diameters^41^; however, the impact on models that assume stick diffusion would likely be small only if the acquisition had a long enough diffusion time.^42^, ^43^ Previously proposed physical phantoms include a variety of pore sizes such as pore diameters of 1.5 mm in glass capillaries,^17^ about 27 µm in hemp fiber,^18^ and about 10 µm in hollow electro‐spun fibers.^22^ Preliminary simulations (Supporting Information Figure S1) suggest that at diffusion times larger than about 100 ms, measured diffusion behavior changes minimally. Nevertheless, the protocol introduced by this study produces long, straight pores that have a limited effect on diffusion along their path. Also, there was a small difference in pore diameter between measurements using microscopy and micro‐CT, which can likely be explained by the microscopy images resolving more small pores due to their finer resolution (~0.32 µm vs 1.65 µm).

### 4.2 Phantom versus human diffusion characteristics

The observed DKI metrics correspond well to those seen in real white matter but have some notable differences. The phantoms were scanned at room temperature, which has a lower diffusivity of free water (~0.0022 mm^2^/s) than human body temperature (~0.003 mm^2^/s).^44^ This difference in the diffusivity of free water means that the diffusivity DKI metrics have a higher maximum possible value in vivo, which should be accounted for when analyzing those metrics. The scan protocol also differed from typical clinical scanner acquisitions, with much shorter δ and Δ times. Given the nonnegligible diameters of the phantoms’ pores, these short diffusion times increase the measured RD and decrease the measured fractional anisotropy (FA) relative to what would be observed at longer diffusion times.

The AD in the phantoms was close to the diffusivity of free water at room temperature, whereas coherently organized white matter in the brain typically has an AD of about 0.001 mm^2^/s, approximately a third of the diffusivity of free water at body temperature.^45^ This discrepancy indicates that the uniform fiber orientation and homogeneous microstructure of the phantoms results in a simpler axial diffusion environment than that observed in the brain. The AK in the phantoms was close to zero, while practically all white‐matter regions in the brain have nonzero AK. This finding further supports the conclusion that there is an anatomically unrealistic homogeneity in the phantoms’ microstructure; real tissue is more heterogeneous and structurally complex than the 3AM phantoms.

The RD in the 3AM phantoms was much lower than the diffusivity of free water at room temperature, indicating that diffusion is hindered and/or restricted perpendicular to the axis of the pores. The mean RK in the nominal 3AM phantom is nonzero, but lower than typical values in orientationally coherent white‐matter tracts.^46^ This indicates that the fibrous pores of the 3AM phantom restrict diffusion but do not recreate the structural heterogeneity of real white matter. The increased pore diameters in 3AM phantoms compared with typical axon diameters mean that RD and RK will change with varying diffusion time; preliminary simulations suggest an RD decrease to 81% of the original value and RK increase to 135% of the original value for a doubling of diffusion time from 13 ms to 26 ms (Supporting Information Figure S1).

The MD in the 3AM phantoms was about 63% of the diffusivity of free water at room temperature, whereas MD in real white matter is typically less than 33% of the diffusivity of water at body temperature. Partially due to the near total lack of restriction or hindering of diffusion in the axial direction, there is less restriction/hindering of diffusion overall in 3AM phantoms than in white matter. The mean kurtosis in the 3AM phantoms was about half the typical values in human white matter,^46^ further indicating that 3AM phantoms have less heterogeneous microstructure than real axonal tracts.

The FA is within the range of typical values observed in the human white matter,^45^ but lower than that observed in the most coherent regions like the corpus callosum.^47^ The micro‐CT images show gaps between adjacent lines of material even at 100% infill density, which leads to a nonnegligible free‐water compartment within the phantoms of 34%. This free‐water compartment reduces the overall FA due to partial voluming, which suggests that a higher FA may be achievable with an altered 3D printing process, as described in section 4.1. This free‐water compartment likely also contributes to the relatively low kurtosis values and the diffusivities being relatively close to the free water value.

### 4.3 Phantom reproducibility and stability

Infill density was the only print parameter that had a large effect on any of the DKI metrics, with its greatest effect on FA, RD, and RK. The likely explanation for this effect is that a lower infill density replaces the elastomeric matrix with free water, increasing the effect of partial voluming between the two components. Future work should verify that changing print parameters has no effect on phantom microstructure by performing microscopy on phantoms produced with different 3D print parameters.

The small effect of the other 3D print parameters on the observed DKI metrics suggests that minor variations in print parameters across different 3D printers should not greatly affect the characteristics of the phantoms they print. However, this also means that it is likely not possible to change print parameters to tune a phantom’s diffusion characteristics. That said, it may be possible to tailor diffusion characteristics using different porous filament materials. For example, the PORO‐LAY filament line contains several filament types that have a porous microstructure that is created by PVA dissolving away, but they differ in the composition of the elastomer, including its hardness level. The low coefficient of variation across identical phantoms supports the conclusion that the 3AM phantom production protocol is repeatable, at least across multiple prints on a single printer with the same material.

Despite that the long‐term stability of the matrix is not available through the manufacturer, the stability analysis in this work showed only small changes over the time period of 2.5 months (Figure 7). Although the scanner is located in a temperature‐controlled facility and it is unlikely that there were substantial temperature changes, it is possible that variation in these temperatures could explain the downward trend in AD over time. Nevertheless, the demonstrated stability of the phantom microstructure over a relatively long time period suggests that one prepared phantom sample can at least be transported to multiple sites in multicenter studies without concern for parameter changes between scans.

### 4.4 Method advantages

The phantoms we have introduced are produced with FDM 3D printing, a widely accessible and inexpensive production technique. This potentially allows customizability of phantoms’ microstructural directionality to create crossing fiber bundles in multiple directions. A single spool of printing material (~$50 USD) can be used to produce hundreds of phantoms. Printing 3AM phantoms requires no specialized FDM features, so it is likely that any FDM 3D printer can be used to produce 3AM phantoms; however, the microstructural properties of the phantoms may depend on the printer. Despite this, the microstructural properties are not expected to change between prints using the same printer. The development of this technique lowers the barrier to entry for researchers to conduct phantom studies for validation of certain dMRI models of white matter. Such studies bridge the gap between simulations and studies using fixed tissue, serving as a useful option for the dMRI modeling community.

Examples of studies that could be conducted with 3AM phantoms may include testing the accuracy of dMRI models and representations in capturing the phantom’s distribution of radii and diffusion time dependence, respectively. Also, changing the within‐layer pattern/directionality of material from layer to layer could simulate crossing fiber bundles, and the effect of crossing fibers on dMRI models and representations could be tested. Furthermore, the ability to change the proportion of water in the phantoms by varying the infill density could be used to simulate various levels of CSF partial volume effects in white‐matter studies. Finally, if phantoms were produced at a larger size, more complex fiber patterns could potentially be designed and used to assess tractography approaches on a common phantom, similar to the approach of the Fiber Cup,^48^ but with a phantom that could be produced to the same specification at multiple sites, so that one phantom need not be transported to different sites.

Compared with existing phantoms, the primary advantage of 3AM phantoms is that they can be manufactured without specialized equipment. The 3AM phantoms complement plain fiber phantoms by providing a diffusion environment analogous to intra‐axonal diffusion. Furthermore, 3AM fibers do not require external frames or molds to manipulate the arrangement of their fibrous pores, like plain fiber phantoms or extruded hollow fiber phantoms do. This ease of production comes with the limitation that the pore diameter distribution is not customizable and that it produces pores with a larger diameter than real axons.

Although the phantoms used for this study are much smaller than typical clinical FOVs, 3AM phantoms could, in principle, be produced at a larger size. This would likely require a much longer time for the PVA to dissolve, which could potentially be surmountable, for example, by designing small holes into the larger phantoms.

The analyses performed for this study show the existence of axon‐mimetic fibrous pores in 3AM phantoms that modulate dMRI signal to approximate white‐matter anatomy. By analyzing the microstructure of the phantoms with both confocal microscopy and phase‐contrast micro‐CT, we have confirmed that the fibrous pores in our phantoms are of an appropriate size and shape to mimic axonal fibers. These findings are supported by the observation of nonzero FA and RK. Furthermore, the quantitative T_1_ and T_2_ scans indicate that the phantoms do not shorten relaxation times enough to significantly reduce SNR in dMRI scans, and they can be reduced by doping (eg, copper II sulphate) to better agree with values found in tissue.

The FDM offers the flexibility to alter the print‐head direction between layers, which may enable the exploration of crossing fiber effects and their associated models. In our preliminary investigations, FA reductions are observed with increasing fiber crossing angle. Future work will investigate optimal phantom design for the validation of crossing fiber models.

## 5 CONCLUSIONS

In this study, we introduce 3AM phantoms, a novel class of dMRI phantoms that are affordable and straight‐forward to produce. The 3AM phantoms mimic the microporous structure of axon bundles in white matter, and the use of 3D printing opens the door for inexpensive and easy‐to‐produce phantoms.

## Supporting information


**FIGURE S1** Radial diffusivity (RD) (A) and radial kurtosis (RK) (B) in simulated cylinders that have diameters with the same gamma distribution as in the phantom (measured using microscopy) versus effective diffusion time
**FIGURE S2** Nominal phantom printing parameters
