# Supplementary material for: Design and characterization of a 3D‐printed axon‐mimetic phantom for diffusion MRI
Source: Magn Reson Med. 2021 Jun 30;86(5):2482–96. doi: 10.1002/mrm.28886 (PMC8596689; doi:10.1002/mrm.28886)
Supplement: Supplementary file 1 — FIGURE S1 Radial diffusivity (RD) (A) and radial kurtosis (RK) (B) in simulated cylinders that have diameters with the same gamma distribution as in the phantom (measured using microscopy) versus effective diffusion time FIGURE S2 Nominal phantom printing parameters [file MRM-86-2482-s001.docx]

**Supporting Information – Manuscript supplementary Figure**


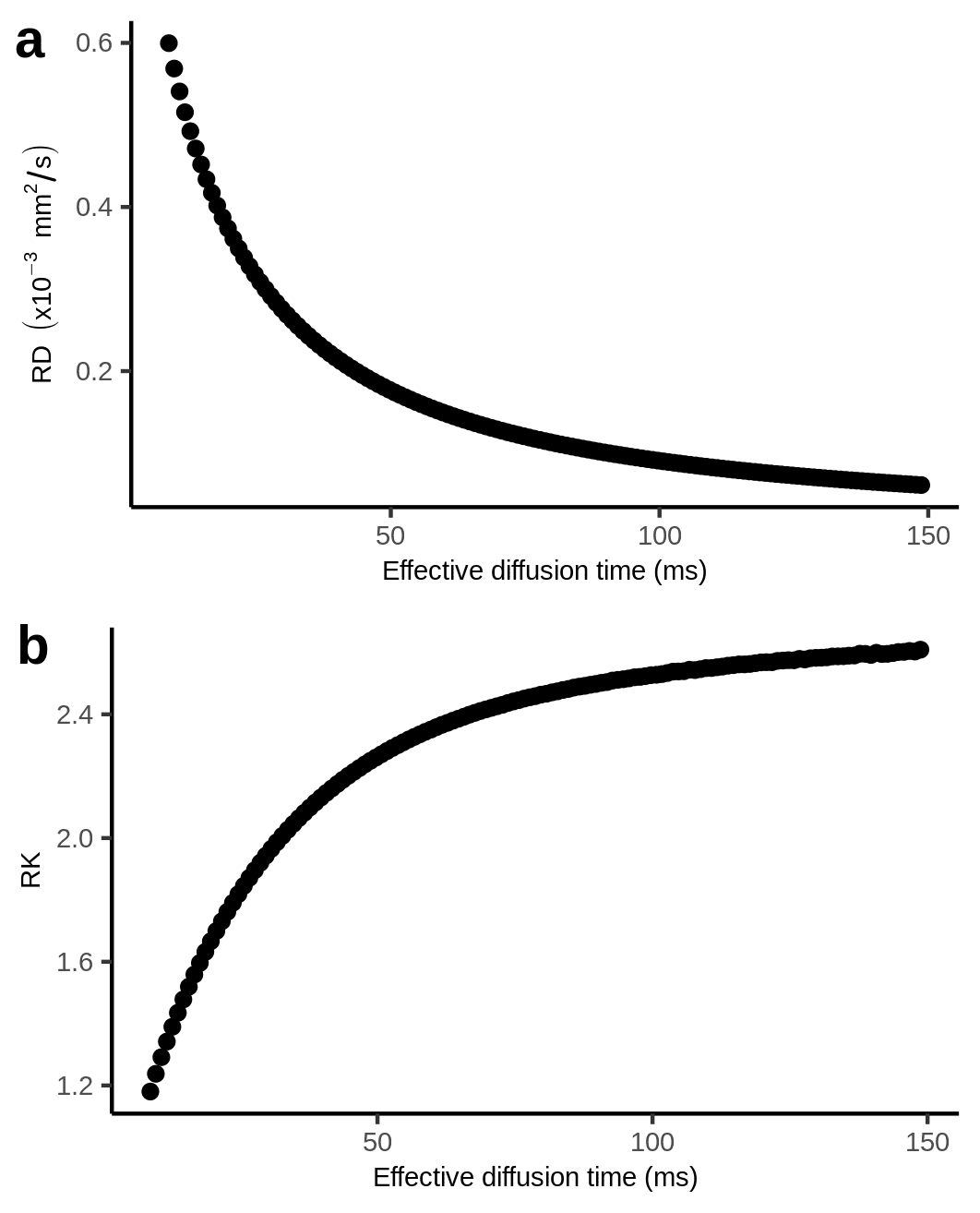


**Supporting information Figure S1.** Radial diffusivity (RD) (a) and radial kurtosis (RK) (b) in simulated cylinders that have diameters with the same gamma distribution as in the phantom (measured using microscopy) vs. effective diffusion time.

**Supporting Information - Production of 3D printed Axon-Mimetic (3AM) phantoms protocol for the validation of diffusion MRI (dMRI)**

Farah N. Mushtaha, Tristan K. Kuehn , Ali R. Khan, Corey A. Baron

*Robarts Research Institute, Western University, London, Canada*

**Abstract**

Validating diffusion MRI (dMRI) representations and models of brain tissue is challenging because there is no reference ground-truth for *in vivo* scans. We describe a form of 3D printed phantoms as a flexible paradigm for investigating and validating microstructural indices and crossing fibres that is reproducible with inexpensive materials.

**Keywords**

Diffusion MRI , Phantoms, Validation

**Figures**

| **Printing parameter** | **Nominal value** |
| --- | --- |
| Infill density | 100% |
| Printing temperature | 225℃ |
| Printing speed | 30 mm/s |
| Layer height | 0.1 mm |

**Supporting information FigureS.2.** Nominal phantom printing parameters .

**Introduction**

Diffusion MRI has the potential to quantify histological features of the brain at a micrometre scale ^1^. However, it is difficult to validate diffusion MRI techniques because there is usually no well-characterized “ground truth” for comparison. Many phantoms with independently verifiable microstructural characteristics have been proposed, but many of them have time-consuming or non-trivial preparation processes, and have limited ability to mimic bundles of crossing fibres ^2^.

We propose a novel phantom produced using fused deposition modeling (FDM) 3D printing with a composite material consisting of rubber-elastomeric polymer and a PVA component (PORO-LAY ^3^). When immersed in water the PVA dissolves, leaving behind small water filled pores with anisotropy along the direction of motion of the print head. These inexpensive and reproducible phantoms can mimic the diffusion characteristics of axons and be produced with complex orientations. This protocol provides a recipe for production and preparation of 3D printed Axon-Mimetic (3AM) phantoms for dMRI validation.

**Materials and equipment**

- Preparation for dMRI scan
- GEL-LAY filament
- Surfactant
- Test tubes
- Airtight test tube stoppers
- Clear plastic container
- Deionized water
- Phantom cylinder STL file [osf.io/dz8rf/](https://osf.io/dz8rf/)
- FDM 3D printer (must be compatible with Cura software)
- Vacuum chamber
- Cage with individual chambers for each phantom.

**Safety**

- Handle the phantom after being printed with gloves at all times.

**Storage**

- Gel-Lay filament
- The quality of the Gel-Lay printing filament is affected by moisture in the air. The spool of Gel-Lay must be kept in a sealed bag with packets of silica gel after use. Silica gel acts as a dessicant that absorbs the moisture in the space it is contained in.
- 3D printed phantoms
- While the phantoms can be immersed in tap water during the dissolving phase, they must be immersed in distilled water when performing the MRI scans.

**Preparing phantoms for dMRI scans procedure**

| 1. For each phantom, use Cura to load the STL file, ensure the nominal print parameters are selected as in Table 1, and choose your desired infill pattern. |
| --- |

1. Arrange the phantoms in the cage, recording the position of each..
2. Place the cage with phantoms in a clear plastic container filled with room temperature tap water. It may be necessary to place a weight on the cage to prevent it from floating to the surface.
3. Let the phantoms dissolve for 7-10 days, refreshing the water every day to avoid saturation and PVA build up.
4. Dump all the tap water and fill the clear plastic container with deionized water
5. Add 1/20th part surfactant to the deionized water.
6. Place the plastic container in the vacuum chamber, and run the vacuum pump until it reaches 1 bar pressure.
7. Leave the phantoms in the vacuum chamber for 48 hours.
8. Prepare test tubes by labelling them and rinsing them with deionized water.
9. While keeping everything underwater, move the phantoms from the cage into the test tubes.
10. Seal the test tube with a stopper and remove it from the container.

NOTE: It is crucial that after the phantoms are out of the vacuum chamber, they stay underwater and don’t come into contact with air.

## **Acknowledgements**

This work was supported by the Canada First Research Excellence Fund, Brain Canada, and Discovery Grants from the Natural Sciences and Engineering Research Council (NSERC).

## **References**

1. Alexander D.C, Dyrby T.B, Nilsson M, and Zhang H. Imaging brain microstructure with diffusion MRI: practicality and applications. *NMR in Biomedicine*. 2017;e3841. doi:10.1002/nbm.3841.
2. Fieremans E. and Lee H.-H. Physical and numerical phantoms for the validation of brain microstructural MRI: A cookbook. *NeuroImage*. 2018;182:39–61.
3. Abu-Sardanah S.O, Hussain U, Moore J, Baron C.A, Peters T.M., Khan A.R. Design and evaluation of a diffusion MRI fibre phantom using 3D printing. *Proc. SPIE*. 10573, 2018.
